# Ultrasound findings in critical care patients: the “liver sign” and other abnormal abdominal air patterns

**DOI:** 10.1186/s13089-016-0039-7

**Published:** 2016-03-11

**Authors:** Joseph Dahine, Annie Giard, David-Olivier Chagnon, André Denault

**Affiliations:** Department of Intensive Care, Université de Montréal, Montreal, QC Canada; Department of Emergency Medicine, Hôpital Sacré-Coeur de Montréal, Montreal, QC Canada; Department of Radiology, Centre Hospitalier de l’Université de Montréal, Montreal, QC Canada; Department of Anesthesiology and Division of Critical Care, Montreal Heart Institute, Université de Montréal, 5000 Belanger Street, Montreal, QC H1T 1C8 Canada; Division of Critical Care, Centre Hospitalier de l’Université de Montréal, Montreal, QC Canada

**Keywords:** Ultrasound GI, Critical care/shock, Diagnostic imaging, Echo, Gastroenterology diagnosis

## Abstract

In critical care patients, point of care abdominal ultrasound examination, although it has been practiced for over 30 years, is not as widespread as its cardiac or pulmonary counterparts. We report two cases in which detection of air during abdominal ultrasound allowed the early detection of life-threatening pathologies. In the first case, a patient with severe *Clostridium difficile* was found to have portal venous gas but its significance was confounded by a recent surgery. Serial ultrasonographic exams triggered a surgical intervention. In the second case, we report what we call the “liver sign” a finding in patients with pneumoperitoneum. These findings, all obtained prior to conventional abdominal imaging, had immediate clinical impact and avoided unnecessary delays and radiation. Detection of abdominal air should be part of the routine-focused ultrasonographic exam and for critically ill patients an algorithm is proposed.

**Electronic supplementary material:**

The online version of this article (doi:10.1186/s13089-016-0039-7) contains supplementary material, which is available to authorized users.

## Background

**Fig. 1:**
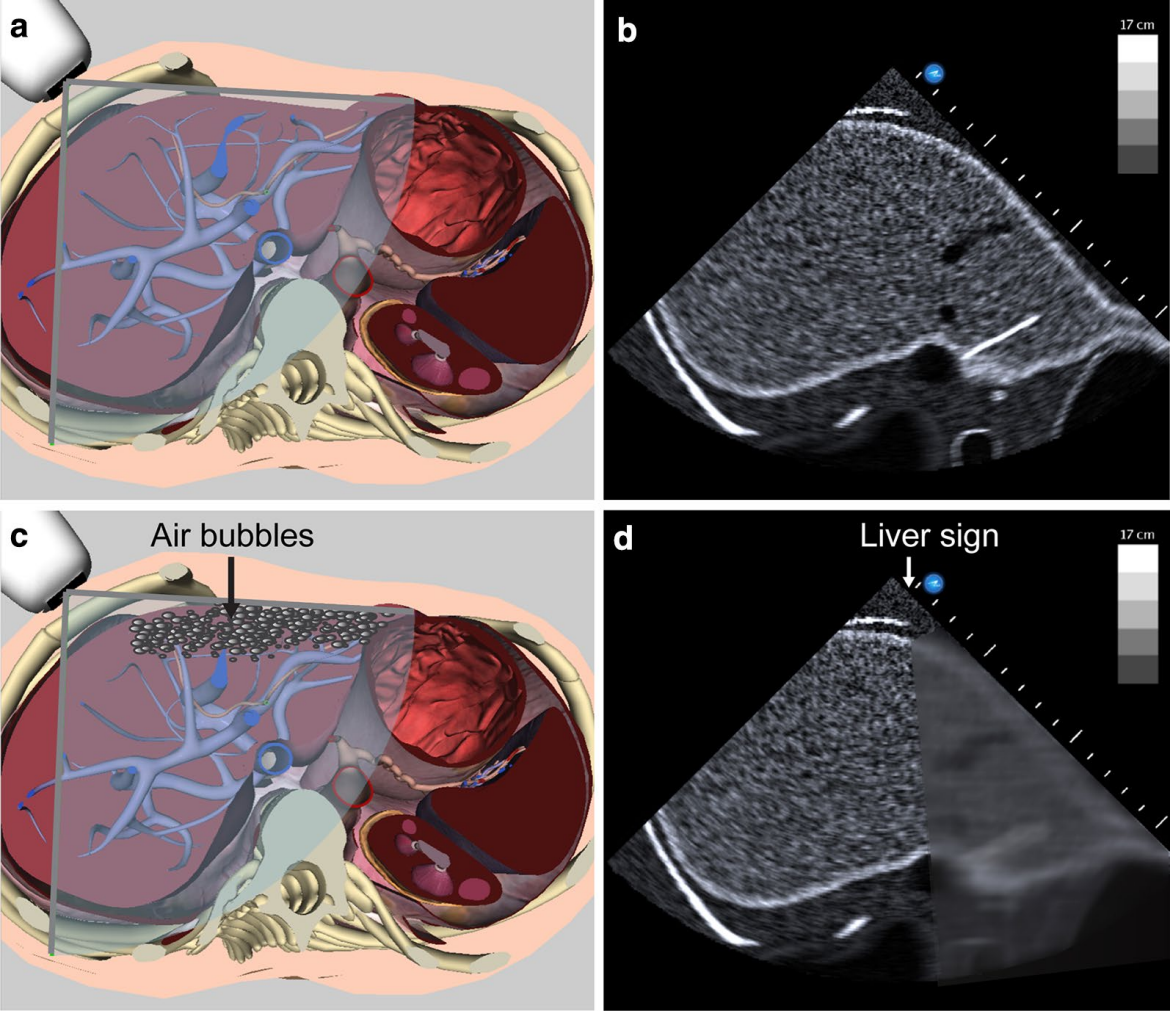
Pneumoperitoneum. **a**, **b** Normal aspect of the liver using a transverse view at the anterior axillary line. **c** When air is present in the abdomen, it will typically accumulate in the non-dependant region just anterior to a portion of the liver. **d** When the ultrasound beam is positioned over this area, the normal acoustic aspect of the liver will be followed by a sharply demarcated acoustic shadow. The term “liver sign” can be used to describe this abnormal air-liver interface. Anatomical and two-dimensional plane using the Vimedix simulator with permission of CAE Healthcare, Ville St-Laurent, QC, Canada

In 2009, the American College of Chest Physicians (ACCP) and La Société de Réanimation de Langue Française (SRLF) proposed guidelines in critical care ultrasound [1]. Those guidelines included not only cardiac but also general critical care ultrasound. The latter included pleural, lung, vascular, and abdominal imaging. Among the technical and cognitive elements required for competence in abdominal ultrasonography, there was no mention of detecting abnormal air in specific areas despite the significant importance of this finding in critically ill patients. Figure 1 illustrates the mechanism of a typical artifact associated with a pneumoperitoneum underlying the liver. Moreover, abdominal ultrasound is a skill that has been practiced for over 30 years in the acute care setting [2, 3]. But over the years, other modalities, such as the focused assessment with sonography in trauma (FAST), have rather gained prevalence in the clinical setting. Given the recent increased availability of point of care ultrasound (POCUS), we submit that perhaps the time has come to include advanced skills as part of the toolbox with which physicians caring for critically ill patients should be familiar. To illustrate this, we report the cases of two patients where such routine use of the focused abdominal ultrasound exam led to the rapid detection of ominous findings of abnormal air patterns as the etiology of the clinical deterioration and expedited care. Written informed consent was obtained from the two patients.

## Case presentation 1

**Fig. 2:**
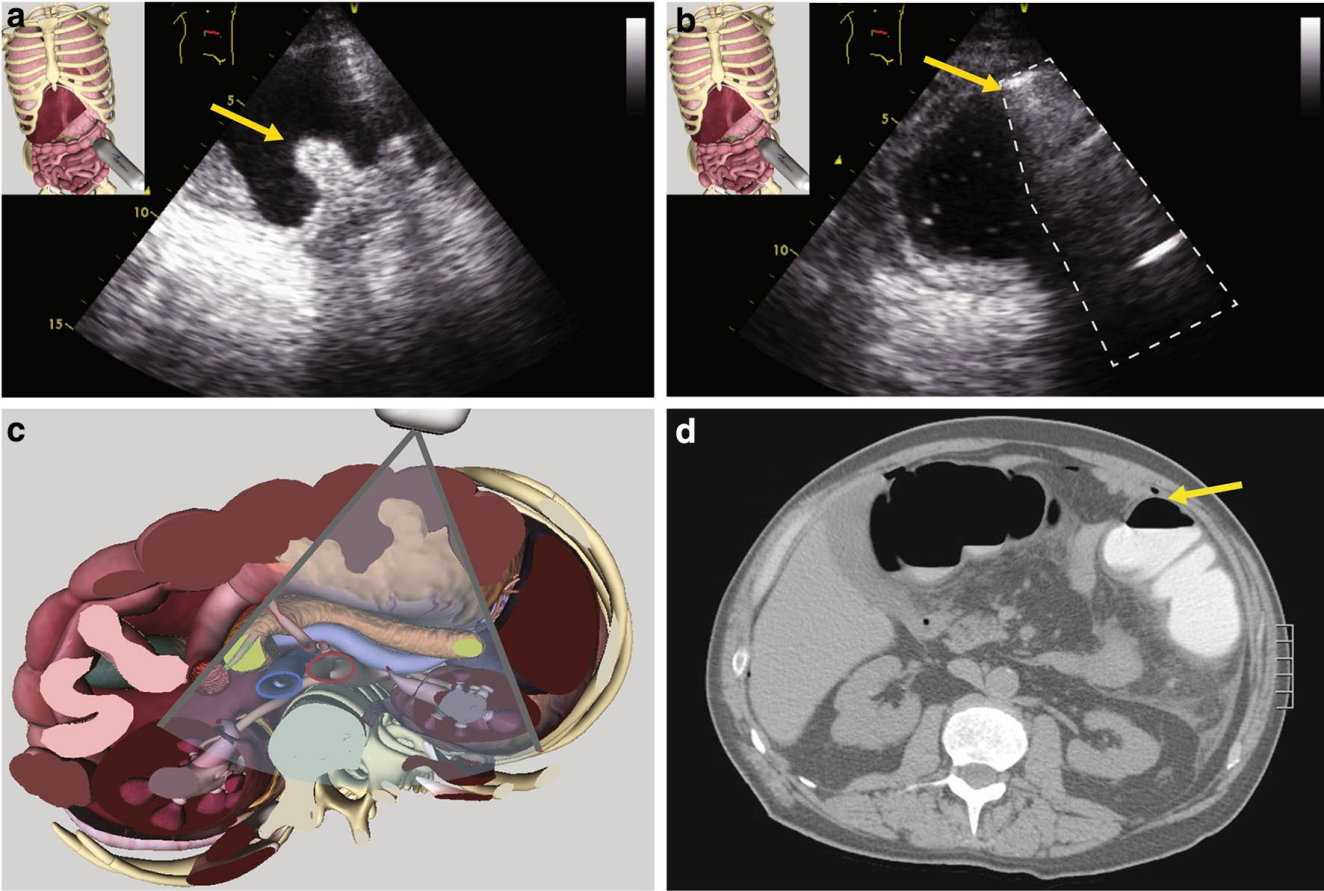
**a** Left upper abdominal transverse view showing an edematous large bowel typical of a «thumb-printing» aspect. The *yellow arrow* denotes the affected area of edematous bowel wall. **b** Air artifact originating from the bowel wall can be seen. The *yellow arrow* shows an echogenic foci in the bowel wall causing shadowing. **c** Corresponding anatomical plane using the Vimedix simulator (CAE Healthcare, Ville St-Laurent, QC, Canada). **d** Computed tomography showing air within the large bowel wall (*yellow arrows*). (See Additional file 1: Video #S1a and Additional file 2: Video #S1b)

**Fig. 3:**
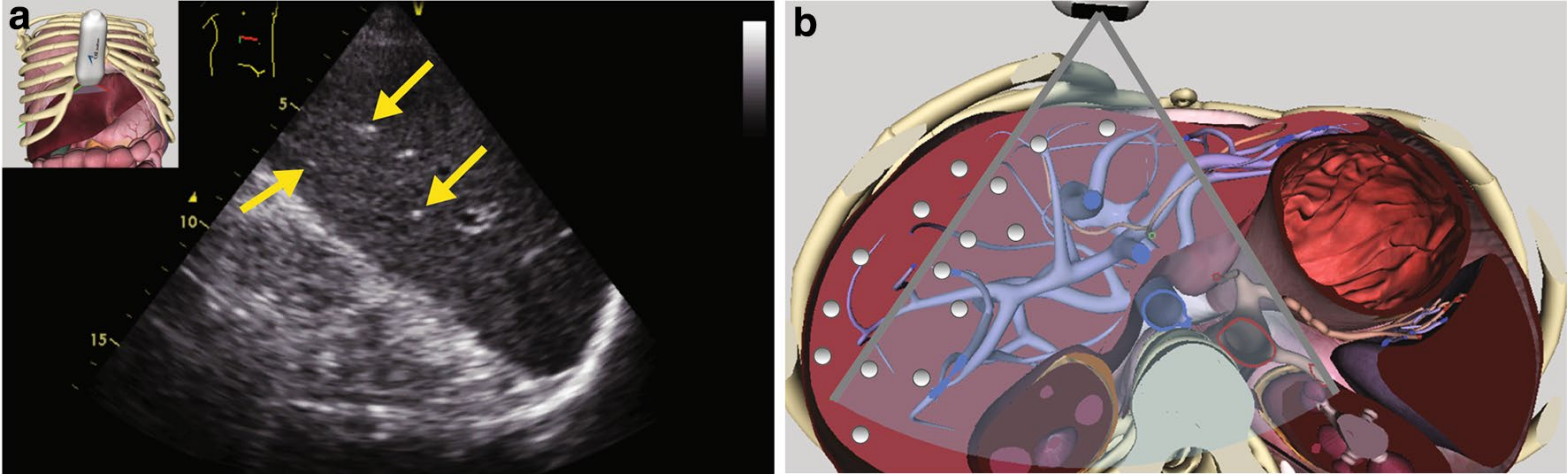
**a** Right paramedian transverse scan of the liver. Numerous small, round, hyperechoic, punctuate air artifact are seen in the periphery of the liver tissue. **b** Corresponding anatomical plane using the Vimedix simulator (CAE Healthcare, Ville St-Laurent, QC, Canada). (See Additional file 3: Video #S2)

A 66-year-old man presented with a 24-h history of severe nausea and abdominal pain. His medical history was remarkable for a rectal adenocarcinoma that was resected 4 months prior to presentation and had left him with a temporary ileostomy. He was otherwise healthy and took no medications. He underwent closure and reanastamosis of his ileostomy 5 days prior to his presentation. Upon arrival to the emergency department, his blood pressure (BP) was 80/40 mmHg and was tachycardic but displayed normal mentation. His examination was remarkable for diffuse abdominal pain and rebound tenderness, but no guarding. Laboratory work revealed acute kidney injury with a creatinine of 180 mmol/L. His pre-operative baseline value was 66 mmol/L. However, his arterial lactate and white blood cell counts (WBC) were normal. The patient received initial fluid resuscitation and underwent a computed tomography scan that showed a dilated cecum (8 cm) and an edematous colon. A *Clostridium difficile* toxin assay returned positive. He was admitted to our intensive care unit (ICU) for monitoring and medical therapy with intravenous metronidazole and oral vancomycin. The patient improved from a hemodynamic, renal as well as abdominal pain point of view, but his WBC and creatinine started to increase 3 days later without new hemodynamic compromise. An abdominal ultrasound (US) was performed and revealed evidence of thumbprinting (Fig. 2a), with the thickened colon wall measured at 17 mm (normal values: <3 mm when measured with high-frequency linear array probes) [4], as well as pneumatosis intestinalis (Fig. 2b) In addition, peripherally located air speckles that follow the portal circulation were present (Fig. 3). These findings were absent on the baseline US exam we performed upon admission. An abdominal X-ray as well as a repeat computed tomography (CT) scan were obtained to confirm the findings (Fig. 2d). The scan was performed with oral but not intravenous contrast in the context of acute kidney injury. A diagnosis of failure of medical therapy was made and a discussion with the surgical team led to a tentative colectomy-saving procedure, namely a redo-ileostomy in order to irrigate vancomycin locally. The patient improved within the next 48 h and was discharged from our ICU.

## Case presentation 2

**Fig. 4:**
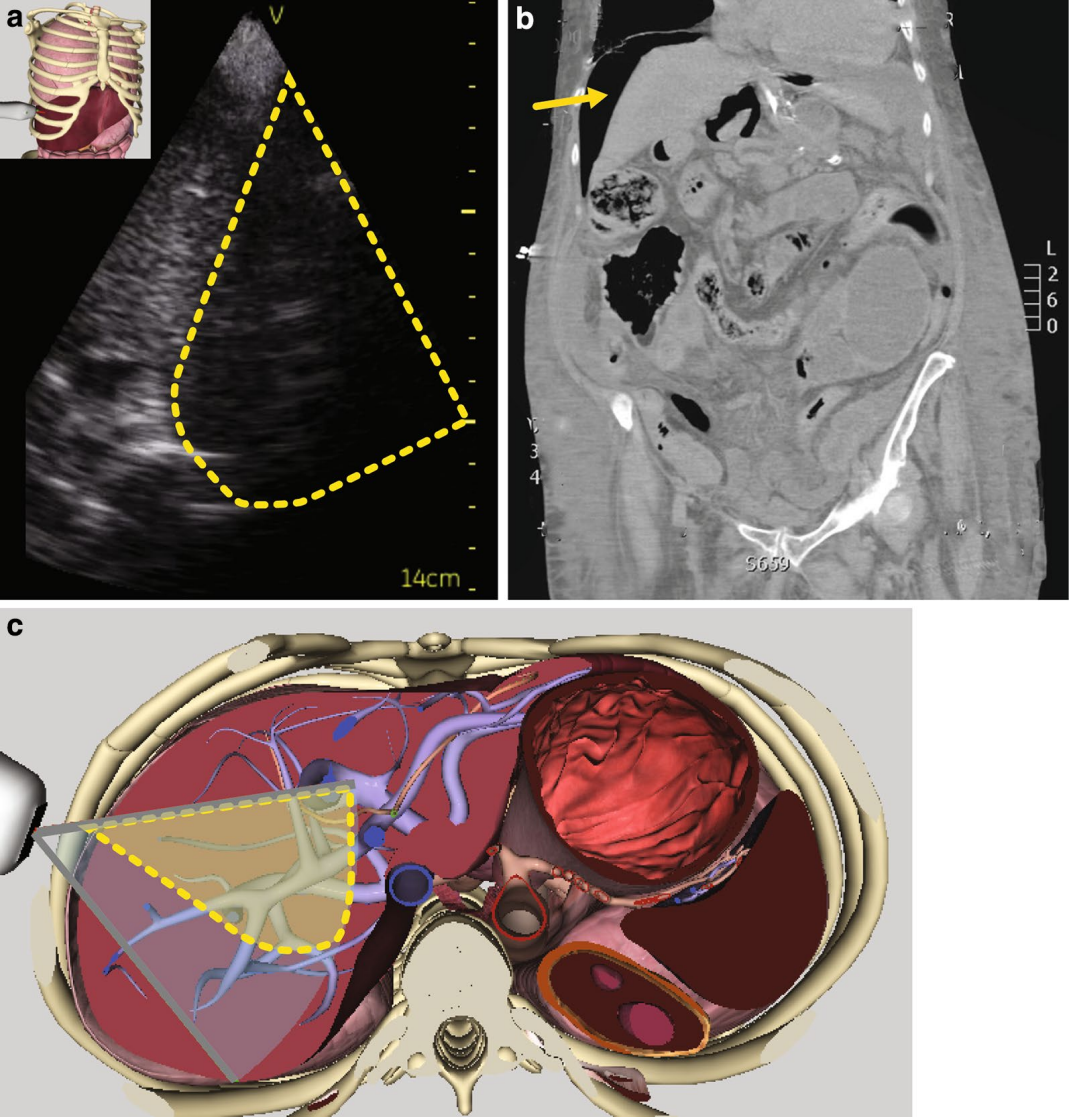
**a** Mid-axillary transhepatic transverse view of the liver in a patient with acute pneumoperitoneum. Note the acoustic shadowing in the *right-sided portion* of the liver which corresponds to the most anterior part of the liver where air will accumulate. **b** Confirmation was obtained with computed tomography. **c** Corresponding anatomical plane using the Vimedix simulator (CAE Healthcare, Ville St-Laurent, QC, Canada). (See Additional file 4: Video #S3)

The ICU team was called for immediate assessment of a 61-year-old woman who was hospitalized on the surgical ward with a complaint of desaturation and acute abdominal pain following dilation of a known anal stenosis which occurred 5 days prior. She was otherwise known for multiple abdominal surgeries in the past, ischemic cardiomyopathy with a 45 % ejection fraction, dyslipidemia and history of drug abuse. At the time, she was known to be taking pregabalin, hydromorphone, acetaminophen, aspirin, atorvastatin, and prophylactic subcutaneous heparin. Upon initial assessment, she was afebrile, tachypneic, and normotensive. Her heart rate was 110/min, BP normal but her oxygen saturation was 85 % on 50 % inspired oxygen. Initial examination was aimed at ruling out an acute coronary syndrome with subsequent congestive heart failure, but an electrocardiogram was normal and a focused transthoracic echocardiography (TTE) did not reveal new wall motion abnormalities or a worsening in the cardiac function. As part of our routine ultrasound examination, we looked at the inferior vena cava (IVC) [5, 6] and portal vein in order to determine her fluid status [7–9]. In order to do this, we usually proceed with our probe in the longitudinal axis, starting midline and anteriorly in the abdomen and moving laterally towards the posterior-axillar line. Surprisingly, we discovered an air artifact that obstructed our view as we were attempting to scan the liver in this motion (Fig. 4a). This was unexpected, as this is a finding compatible with pneumoperitoneum, which is not expected as a complication of anal dilation, given that the rectum is a retroperitoneal structure. We hence performed an upright abdominal film that confirmed free air under the diaphragm as well as a CT scan without contrast (Fig. 4b) to document the findings. This information guided our surgeons who took the patient back to the operating room (OR). The findings intraoperatively were that of fecal peritonitis secondary to an anterior faux-conduit that was created iatrogenically by the initial anal dilation attempt and communicated with the peritoneum. The patient had an unremarkable post-op course and was discharged from our ICU.

## Conclusions

In the above cases, we used the ultrasound to observe three abnormal air patterns that should alert the clinician to an intra-abdominal pathology, namely intraperitoneal air (case#2), portal venous gas (case#1), and intramural air (case#1).

Intraperitoneal air can be seen using the liver as an acoustic window. Indeed, the liver offers a unique echogenic window. Although the hepatic flexure of the colon can sometimes be found interposed, there is generally no bowel that creates artifact in the right upper quadrant. Free air will accumulate anteriorly in the supine position and create an air artifact interfering with the normal liver texture (Fig. 1). It is of importance not to press deeply with the ultrasound probe as this can shift small amount of free air away from the US field. This phenomenon of air interposition should be considered abnormal and is similar to the “lung point” shadow that occurs in a pneumothorax where ultrasound reveals loss of normal lung sliding [10]. We propose the term “liver sign” to describe this longitudinal band air artifact that interrupts normal scanning of the liver from the anterior abdominal midline to the posterior axillary in the longitudinal axis.

Traditionally, other studies have described the main ultrasonographic finding of free intraperitoneal air as peritoneal stripe enhancement with associated reverberation artifacts [11, 12] and recommended the use of a linear probe to improve detection [13] similarly to what is recommended in lung ultrasound. Other indirect signs have also been described. Examples include thickened bowel loops (such as in our first case), air bubbles in ascitic fluid and thickening of bowel or gallbladder wall associated with ileus [13]. Another sign is the scissor maneuver, described by Karahan et al. which entails repeatedly applying pressure with the linear ultrasound probe sagitally oriented in the right paramedian epigastric area and looking for a shift in the reverberation (i.e., presence upon releasing probe pressure; disappearance upon increased pressure using the caudal part of the probe) [14].

However, our cases highlight two important points that contrast with the existing literature. First, we did not document a peritoneal stripe enhancement, but the reverberation artifacts were present. Second, the phased array probe was used rather than the typical abdominal convex or linear probe. Several handheld echo devices do not have an abdominal probe. Rather, the phased array probe has become more prominently used as it allows reliable examination of the cardiac structures, which remains at the core of the assessment of the unstable patient. In addition, abdominal, renal, and cranial settings are commonly available when using the cardiac-phased array probe.

Caution has to be exercised in the interpretation of this finding as free air usually resolves rapidly post-laparotomy or laparoscopy but can rarely persist for weeks [15]. In *C. difficile* colitis, such as in our first case, complications such as worsening bowel wall edema, bowel distention and pneumatosis intestinalis can help guide the clinician towards surgical therapy prior to an obvious macrohemodynamic deterioration which would occur after perforation. Furthermore, artifacts that can mimick pneumoperitoneum have been described such as pseudo-pneumatosis intestinalis and pseudo-thickened gut [16]. Interested readers are encouraged to explore this topic further as it is beyond the scope of this article.

Portal air or hepatic portal venous gas can result from mesenteric ischemia or other conditions associated with alteration of the intestinal mucosa [17]. Using sonography, air in the portal vein can be seen as flowing echogenic bubbles mostly in the periphery. In contrast, air in the biliary tree or pneumobilia is commonly seen following biliary instrumentation when the sphincter of Oddi becomes incompetent. It can occur in patients with acute cholecystitis. With ultrasound, reflective linear echoes will be seen. However, they are less mobile, more centrally located, they change with position, are associated with several comet tail artifacts and are not associated with venous flow [18].

Several technical limitations preclude complete visualization of the gastrointestinal tract. Ultrasound itself is operator-dependent. In a small study of 31 patients in the emergency room with abdominal pain in which five patients were found to have pneumoperitoneum by both CT imaging and abdominal X-ray, the sensitivity and specificity of a hand-carried ultrasound were 80 and 81 %, respectively [19]. Hence, clinical suspicion of abdominal pathology without supporting evidence on US should always lead to further imaging by CT scan.

However, a recent study showed that senior physicians with experience in abdominal US can rapidly learn to identify patterns suggestive of pneumoperitoneum with almost 90 % accuracy [20]. The study also indicated that a focused abdominal US exam comprising two views (epigastric and right hypochondrium) was similar in sensitivity and specificity to the full abdominal exam. We believe that this further supports the importance of encouraging exposure to abdominal US in the ICU and to ensure clinicians are trained to not only identify abnormal patterns, but first and foremost to acquire images at the bedside autonomously.

**Fig. 5:**
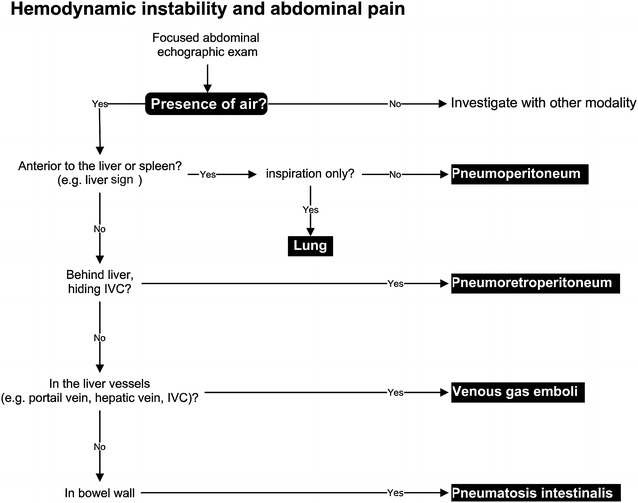
Proposed approach using bedside ultrasound for patients with abdominal pain and hemodynamic instability. If air is present anterior to the liver or spleen (in the absence of a recent abdominal surgery) during both inspiration and expiration, a pneumoperitoneum should be suspected. A pneumoretroperitoneum will be associated with air behind the liver hiding the inferior vena cava (IVC). Venous gas embolism will be associated with air in the liver typically in the portal vein mostly in the peripheral regions of the liver. Air embolism can also be seen in the hepatic vein and the IVC. Finally pneumatosis intestinalis is characterized by air in the bowel lumen

The two cases we report offer additional insight into the usefulness of adding a focused abdominal exam to the already well-established focused TTE as proposed by the ACCP, SRLF, and other organizations [1, 21, 22]. Critical impending complications will be suspected when air is present in specific locations such as the peritoneum, the retroperitoneum, the lumen of preformed organs or vessels (such as the bladder or the portal vein), the parenchyma of organs (such as liver or kidney), and the abdominal wall [12]. Figure 5 summarizes an approach for the identification of normal and abnormal abdominal air.

## Consent

Written informed consent was obtained from the patient for publication of this case report and any accompanying images.

## Additional files

10.1186/s13089-016-0039-7 Pneumatosis–patient view.
10.1186/s13089-016-0039-7 Pneumatosis–simulator view.
10.1186/s13089-016-0039-7 Air in the portal circulation.
10.1186/s13089-016-0039-7 The liver sign.

## Abbreviations

ACCP: American College of Chest Physician
BP: blood pressure
CT: computed tomography
FAST: focused assessment with sonography in trauma
ICU: intensive care unit
IVC: inferior vena cava
OR: operating room
POCUS: point of care ultrasound
SRLF: La Société de Réanimation de Langue Française
TTE: transthoracic echocardiography
US: ultrasound
WBC: white blood cell counts
